# NES1/KLK10 gene represses proliferation, enhances apoptosis and down-regulates glucose metabolism of PC3 prostate cancer cells

**DOI:** 10.1038/srep17426

**Published:** 2015-11-30

**Authors:** Jiajia Hu, Hu Lei, Xiaochun Fei, Sheng Liang, Hanzhang Xu, Dongjun Qin, Yue Wang, Yingli Wu, Biao Li

**Affiliations:** 1 Department of Nuclear Medicine, Rui jin Hospital, School of Medicine, Shanghai JiaoTong University; 197 Ruijin Second Road, Shanghai 200025, China; 2 Department of Pathophysiology, Key Laboratory of Cell Differentiation and Apoptosis of Chinese Ministry of Education, School of Medicine, Shanghai JiaoTong University; 280 Chongqing South Road, Shanghai 200025, China; 3 Department of Pathology, Rui jin Hospital, School of Medicine, Shanghai JiaoTong University; 197 Ruijin Second Road, Shanghai 200025, China; 4 Department of Nuclear Medicine, Xin hua Hospital, School of Medicine, Shanghai JiaoTong University; 1665 Kongjiang Road, Shanghai 200025, China

## Abstract

The normal epithelial cell-specific-1 (NES1) gene, also named as KLK10, is recognised as a novel putative tumour suppressor in breast cancer, but few studies have focused on the function of KLK10 in human prostate cancer. Our study confirms that the expression of KLK10 in prostate cancer tissue and cell lines (PC3, DU145, and LNCaP clone FGC) is low. Given that the androgen-independent growth characteristic of the PC3 cell line is more similar to clinical castration-resistant prostate cancer, we studied the role of KLK10 in PC3. *In vitro* and *in vivo* assays showed that over-expressing KLK10 in PC3 could decelerate tumour proliferation, which was accompanied with an increase in apoptosis and suppression of glucose metabolism. The related proteins, such as Bcl-2 and HK-2, were down-regulated subsequently. Furthermore, by up-regulating Bcl-2 or HK-2 respectively in the PC3-KLK10 cell line, we observed a subsequent increase of cell proliferation and a synchronous up-regulation of HK-2 and Bcl-2. Besides, KLK10 expression was also increased by Bcl-2 and HK-2, which suggests that there is a negative feedback loop between KLK10 and Bcl-2/HK-2. Thus, our results demonstrated that KLK10 may function as a tumour suppressor by repressing proliferation, enhancing apoptosis and decreasing glucose metabolism in PC3 cells.

Prostate cancer is the most commonly occurring cancer in men in the developed countries, and the second frequently diagnosed cancer worldwide^1^. China is one of the countries with a low incidence of prostate cancer. Data from the Global Estimates indicated that the age standardised incidence rate of prostate cancer in China is 4.3 per 100,000, which is much lower than the rates in European and American countries^2^. However, with population aging and the western changes in lifestyle and dietary structure of Chinese people, a Chinese retrospective population-based cohort study indicated a remarkable increase in the incidence rate of prostate cancer between 2000 and 2009 in Shanghai^3^. Surgical and hormonal therapies have shown beneficial effects for early-stage, hormone-responsive disease. However, given the non-specific manifestation of prostate cancer, most patients are diagnosed in the advanced stages when surgery is inappropriate. Endocrine therapy is an important prostate cancer treatment, but it is not 100% effective. When the disease progresses, tumours become resistant to castration and no longer respond to hormonal deprivation therapies, and few treatment options are available for more aggressive and even refractory prostate cancer^4^. Thus, the prostate cancer survival rate is disappointingly low^3^. The progression of prostate cancer is regulated by both activation of tumour-promoting genes and inactivation of tumour-inhibiting genes^5^. More effective treatment options for refractory prostate cancer are crucial to develop gene therapy.

The normal epithelial cell-specific-1 (NES1) gene was identified in 1996 by subtractive hybridisation between normal and immortalised breast epithelial cell lines^6^. NES1 cDNA was revealed as a novel serine protease with high homology to the glandular kallikrein family^7^, and the localisation of the NES1 gene is shown on chromosome 19q13.4, a locus where most kallikreins are located^8^,^9^. Based on these characteristics, the NES1 gene is designated as KLK10, a member of the kallikrein family, and its encoded protein is human kallikrein 10 (hK10)^10^. However, the secret protein hK10 is not a functional serine protease^11^. The expression of hK10 in normal human tissues is generally cytoplasmic and not organ-specific, with representative organs being the breast, prostate and kidney^12^. The physiological functions and substrates of KLK10 remain unclear. Previous studies, including in silico analyses, have shown that the expression level of KLK10 is decreased in majority of breast cancer cell lines, whereas transfection of the KLK10 gene into KLK10-negative breast cancer cells can suppress tumour formation in nude mice^8^, thereby suggesting that this gene may function as a novel putative tumour suppressor^13^,^14^. KLK10 mRNA was also found to be down-regulated in prostate cancer cell lines^8^. However, few studies have focused on the function of KLK10 in human prostate cancer.

In the current study, we confirmed that the expression of KLK10 was low in prostate cancer tissue and cell lines, including LNCaP clone FGC and PC3. Both have been widely utilised as cell models for prostate cancer studies and are generally assumed to represent early and late stages of prostate cancer, respectively^15^. The PC3 cell line was established in 1979 from bone metastasis of a grade IV prostatic adenocarcinoma patient^16^, which is more similar to castration-resistant prostate cancer in the clinical situation. Considering the androgen-independent growth characteristics of the PC3 cell line, it was chosen in our study as an advanced prostate cancer model to investigate the effect of KLK10 on cancer proliferation, apoptosis and glucose metabolism.

## Results

### KLK10 expression is low in prostate cancer tissue and cell lines

**Figure 1:**
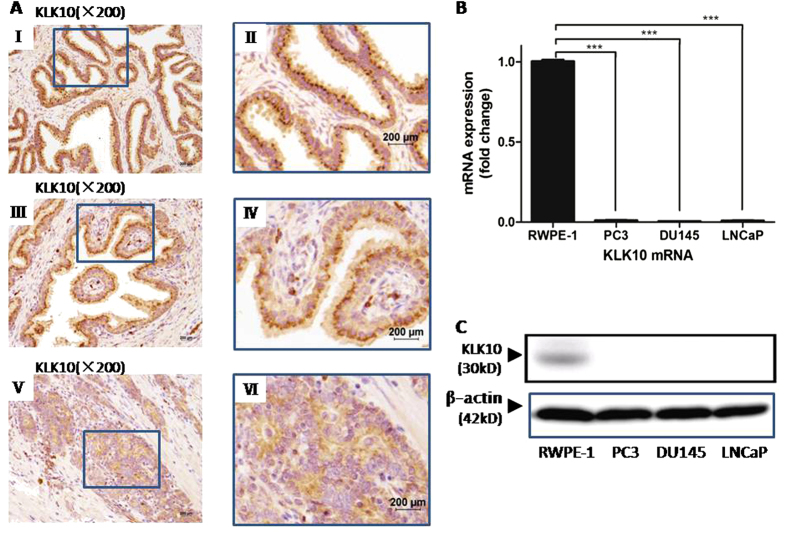
Expression of KLK10 is low in prostate cancer tissue and cell lines. (**A**: I, II) KLK10 protein grains presented brown and were regularly arranged in the cytoplasm of BPH tissue. (**A**: III, IV) KLK10 protein gains presented brown and were regularly arranged in the cytoplasm of matched adjacent normal tissue of prostate cancer. (**A**: V, VI) KLK10 protein grains were lighter or even absent and were irregularly arranged in the cytoplasm of prostate cancer tissue. (**B**) KLK10 mRNA expression was significantly lower in PC3, DU145 and LNCaP clone FGC than in RWPE-1. (**C**) KLK10 protein expression was almost lost in PC3, DU145 and LNCaP clone FGC compared with that in RWPE-1.

In the tissue of benign prostate hyperblastosis (BPH) (Fig. 1A, I and II) and the matched adjacent normal tissue of prostate cancer (Fig. 1A, III and IV), hK10 presented as brown or dark brown grains in the cytoplasm near the nucleus, mainly facing the side of the acinar lumina. However, in prostate cancer tissue, the expression of hK10 was much lower, which presented as light or absent grains, irregularly arranged in the cytoplasm (Fig. 1A, V and VI). The positive rate of hK10 expression in the cancer tissue of prostate cancer patients was only 38.3% (23/60). By contrast, in the matched adjacent normal tissue of prostate cancer patients and in the tissue of BPH patients, the positive rate was significantly higher (93.33% [56/60] and 100% [20/20]; *P* < 0.0001, respectively; Table 1).

In the prostate cancer cell lines (PC3, DU145 and LNCaP clone FGC), KLK10 mRNA and protein expression was significantly lower than that in the normal prostate epithelial cell line (RWPE-1) (Fig. 1B,C). This result was consistent with hK10 expression in prostate cancer tissue. As an androgen-independent prostate cancer cell line, the PC3 cell line was investigated for KLK10 function in prostate cancer.

### KLK10 decelerates PC3 cell proliferation

**Figure 2:**
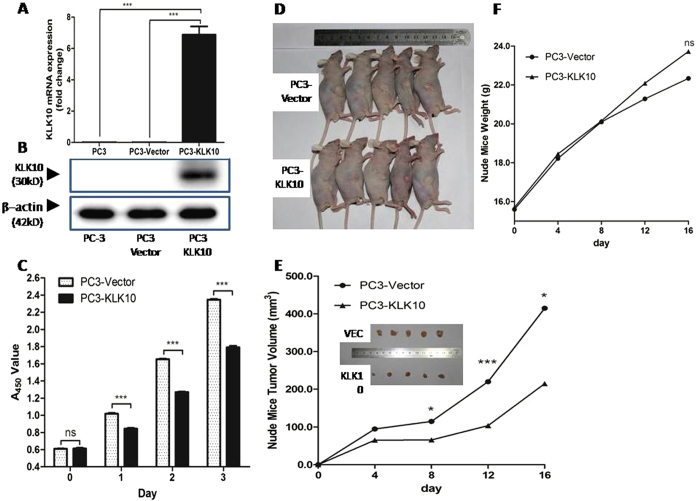
KLK10 gene inhibits the proliferation of PC3 cells. (**A**,**B**) After transducing the KLK10 gene into the PC3 cell line, the mRNA and protein expression levels of KLK10 were significantly higher in PC3-KLK10 than in PC-3 and the blank Vector group. (**C**) CCK-8 cell proliferation assay showing the growth deceleration of PC3-KLK10 cell lines 24 h after cell seeding, and this phenomenon lasted for 3 days (*P* < 0.001). (**D**) General images of PC3-KLK10- and Vector-transplanted tumours showed that the tumour volume was smaller in the KLK10-over-expressing group than in the Vector group. (**E**) Tumour volume line graph showed that tumours were successfully generated in nude mice 4 days after cell transplantation; the PC3-KLK10 tumour grew more slowly than the PC3-Vector tumour, and a significant difference in tumour volume was found between these two groups starting from the eighth day. (**F**) Weight line graph showed that the mouse weight was lower in the PC3-Vector group than in the PC3-KLK10 group from the eighth day, but the difference was not significant (*P* > 0.05).

Using reconstructed lentiviral vector pLVX-KLK10-IRES-ZsGreen1, viral transfection and cell sorting, a high-purity KLK10-expressed stable cell line PC3-KLK10 was obtained. Its KLK10 expression was much higher than that of the PC3-Vector cell line, as confirmed by quantitative real-time PCR (qRT-PCR) and Western blot (WB) (Fig. 2A,B).

To investigate the KLK10 gene’s potential to inhibit cell growth in the PC3 cell line, cell proliferation ability was measured with a CCK-8 assay lasting for 3 days. Given that we utilised the KLK10 over-expressed stable PC3 cell line, we compared the cell growth line between PC3-KLL10 and PC3-Vector cells to analyse the growth difference. As shown in Fig. 2C, the deceleration of PC3-KLK10 cell growth was observed at 24 h after cell seeding (*P* < 0.001).

PC3-KLK10 and PC3-Vector xenograft tumours were generated successfully in nude mice 4 days after cell transplantation. The PC3-KLK10 tumour grew more slowly than the PC3-Vector tumour (Fig. 2D), and a significant difference in tumour volume was observed at 8 days (Fig. 2E). The mice weight of the PC3-Vector group was lower than that of the PC3-KLK10 group, without a significant difference (*P* > 0.05; Fig. 2F), possibly because of the rapid growth of the PC3-Vector tumour.

### ^18^F-FLT micro PET/CT scan confirms a lower proliferation metabolism in KLK10-expressed PC3 tumour, consistent with the lower expression of Ki-67 protein

**Figure 3:**
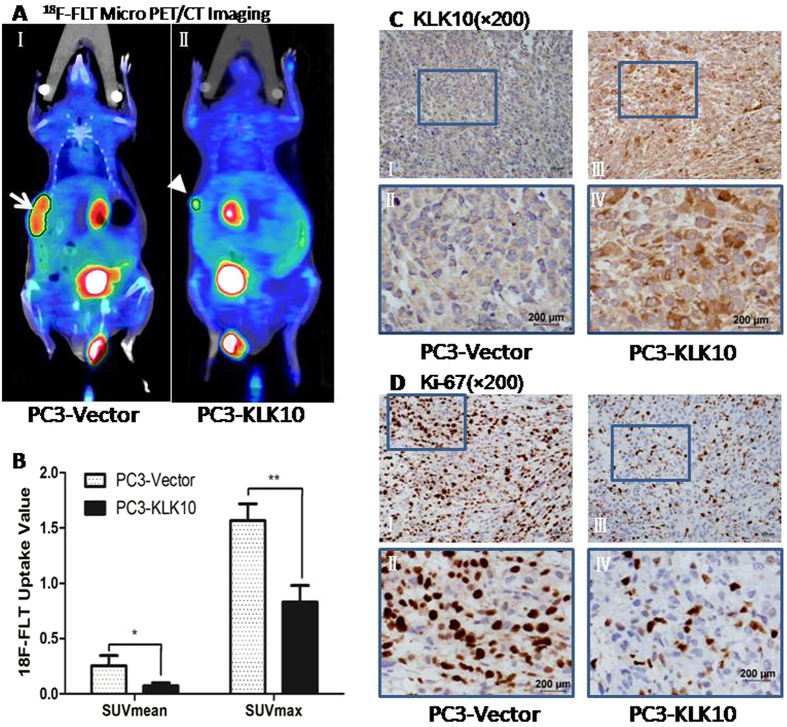
^18^F-FLT Micro PET/CT scan confirmed a low proliferation metabolism in the KLK10-expressing stable PC3 tumour, which is consistent with the low expression of the Ki-67 protein. (**A**: I, II) ^18^F-FLT micro PET/CT scan showed that the ^18^F-FLT uptake of the subcutaneous transplanted tumour was lower in the PC3-KLK10 group (arrow head) than in the Vector group (arrow); in the images, hot spots of ^18^F-FLT can also be found in the digestive and urinary systems, and the background was clean without the interference of other abnormal hot spots. (**B**) Bar chart of ^18^F-FLT uptake semi-analysis showed that the ^18^F-FLT uptake of the PC3-KLK10 tumour, measured as SUV_mean_ and SUV_max_, was significantly lower than that of the PC3-Vector tumour (0.07 ± 0.03 vs. 0.25 ± 0.09; 0.83 ± 0.15 vs. 1.57 ± 0.15)(*P* < 0.05 and *P* < 0.01). (**C**: I-IV) Expression of KLK10 measured by IHC confirmed KLK10 over-expression in the PC3-KLK10-transplanted tumour. (**D**: I-IV) Expression of the Ki-67 protein measured by IHC showed a significant down-regulation in the PC3-KLK10 group compared with the Vector group.

To non-invasively assess the proliferation of xenograft tumours, ^18^F-labelled 3′-deoxy-3′-fluorothymidine (^18^F-FLT) micro positron emission tomography/ computer tomography (PET/CT) scan, using the thymidine analogue ^18^F-labelled 3′-deoxy-3′-fluorothymidine as imaging agent, was conducted to investigate cellular proliferation *in vivo*^17^. ^18^F-FLT is trapped within the cytosol after being monophosphorylated and enters the exogenous DNA pathway as a specific substrate by the action of thymidine kinase 1 (TK1), a principle enzyme in the salvage pathway of DNA synthesis. Therefore, its accumulation is tightly linked to TK1 enzyme activity and the degree of DNA synthesis^18^. In our study, hot spots of ^18^F-FLT were found in subcutaneous tumours and the digestive and urinary systems. The background was clean, without interference from other abnormal hot spots (Fig. 3A). The ^18^F-FLT uptake of PC3-KLK10 tumour, measured as SUV_mean_ (mean standardised uptake value) and SUV_max_ (maximal standardised uptake value), was significantly lower than those of PC3-Vector tumour (0.07 ± 0.03 vs. 0.25 ± 0.09; 0.83 ± 0.15 vs. 1.57 ± 0.15) (*P* < 0.05 and *P* < 0.01; Fig. 3B), which coincided with the proliferation assays *in vitro* and *in vivo*.

Ki-67 protein expression measured by immunohistochemistry showed a significant down-regulation in PC3-KLK10 tumour (Fig. 3C,D), consistent with the results of ^18^F-FLT micro PET/CT scan.

### KLK10 promotes apoptosis of PC3 cells, resulting in the accumulation of the percentage of PC3 cells in the sub-G1 phase with the down-regulation of Bcl-2

**Figure 4:**
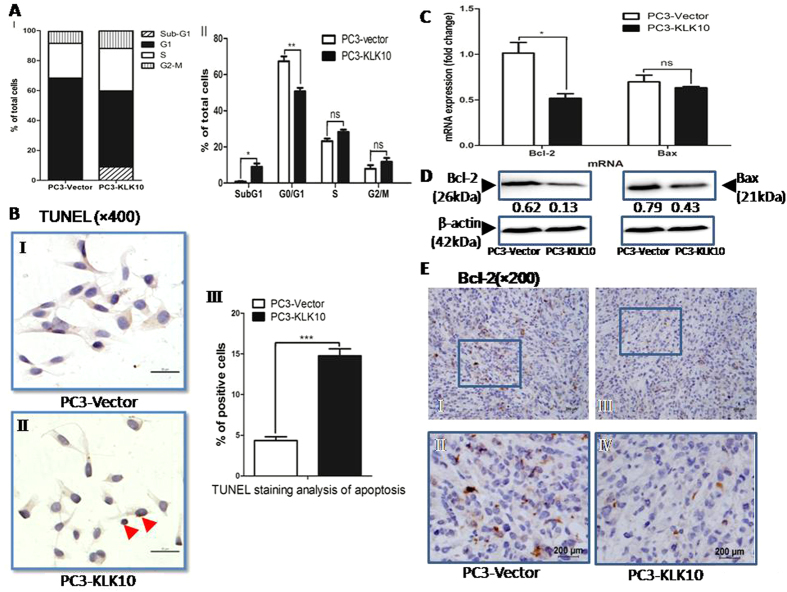
KLK10-induced apoptosis of PC3 cells results in the accumulation of PC3 cells in sub-G1 phase with the down-regulation of Bcl-2. (**A**: I-II) Cell cycle showed a significant accumulation of PC3-KLK10 cells in the sub-G1 phase, indicating apoptosis, compared with PC3-Vector cells (9.01% ± 3.165% vs. 0.82% ± 0.372%, *P* < 0.05). The percentage of PC3-KLK10 cells in the G1 phase was significantly reduced (50.74% ± 3.446% vs. 67.41 ± 4.69%, *P* < 0.01). (**B**: I-III) TUNEL assay staining of apoptotic cells showed obvious positive cells with brown colouring in PC3-KLK10 cell nuclei (arrow head). The apoptotic index of PC3-KLK10 was 14.76% ± 1.950%, which was higher than that of PC3-Vector cells (4.36% ± 1.031%)(*P* < 0.0001). (**C**) qRT-PCR showed the significant down-regulation of Bcl-2 and the slight down-regulation of Bax in the PC3-KLK10 cell line (*P* < 0.05 and *P* > 0.05). (**D**) WB showed the significant down-regulation of the Bcl-2 and Bax proteins in KLK10-over-expressing PC3 cells. (**E**: I-IV) Low expression of the Bcl-2 protein in PC3-KLK10 xenograft tumour tissue measured by IHC confirmed the result of WB.

To test the underlying mechanism that leads to the KLK10-induced deceleration of cell proliferation, we observed the effects of KLK10 on PC3 cells by detecting cell cycle progression and apoptosis. A significant accumulation of PC3-KLK10 cells in the sub-G1 phase, indicative of apoptosis, was observed compared with PC3-Vector cells (9.01% ± 3.165% vs. 0.82% ± 0.372%; *P* < 0.05; Fig. 4A). The percentage of PC3-KLK10 cells was significantly reduced in the G1 phase (50.74% ± 3.446% vs. 67.41% ± 4.69%, *P* < 0.01) but slightly increased in the S phase and G2/M phase without a significant difference (*P* > 0.05; Fig. 4A). Terminal deoxynucleotidyl transferase-mediated dUTP nick end labelling staining of apoptotic cells showed obvious positive brown cells in PC3-KLK10 cell nuclei (Fig. 4B-II). The apoptotic index of PC3-KLK10 was 14.76% ± 1.950%, which was more than that of PC3-Vector cells (4.36% ± 1.031%; *P* < 0.001; Fig. 4B-III). The results showed that KLK10 could promote apoptosis of PC3 cells. Although the percentage of apoptosis in PC3-KLK10 cells was not very high, this phenomenon may explain the deceleration of cell proliferation.

The Bcl-2 gene, a classic and important anti-apoptosis gene, was detected by qRT-PCR and WB. Figure 4C,D showed a down-regulation of Bcl-2 mRNA and protein in the PC3-KLK10 cell line. In the PC3-KLK10 xenograft tumour of nude mice, Bcl-2 protein expression was also low (Fig. 4E). Bax, another important gene and one of the proapoptotic Bcl-2 family proteins, was slightly down-regulated in the PC3-KLK10 cell line (Fig. 4C,D), but similar findings were observed in Vector and PC3-KLK10 xenograft tumour tissues (Supplemental Fig. 1A). Meanwhile, the Bax/Bcl-2 protein ratio in the PC3-KLK10 cell line was still higher than that in Vector (0.43/0.13 vs. 0.79/0.62).

PARP is a caspase-3 substrate, and PARP cleavage has been widely considered as an indicator of apoptosis. Caspase-3 is an important caspase effector that promotes caspase-dependent cell apoptosis. However, the cleaved caspase-3 and PARP proteins in KLK10-expressed PC3 cells were not detected (Supplemental Fig. 1B,C). In addition, caspase-3 enzyme activation was not obvious in PC3-KLK10 cells, as measured by caspase-3 enzyme activity assay (data not shown). These results could be explained by the low percentage of apoptosis. Another potential reason was that the KLK10 gene might inhibit PC3 cell proliferation via the caspase-independent cell apoptotic pathway. The exact mechanism of cell apoptosis will be explored in further experiments.

### KLK10 gene inhibits glucose metabolism of PC3 cells, with lower ^18^F-FDG uptake via the down-regulation of HK-2

**Figure 5:**
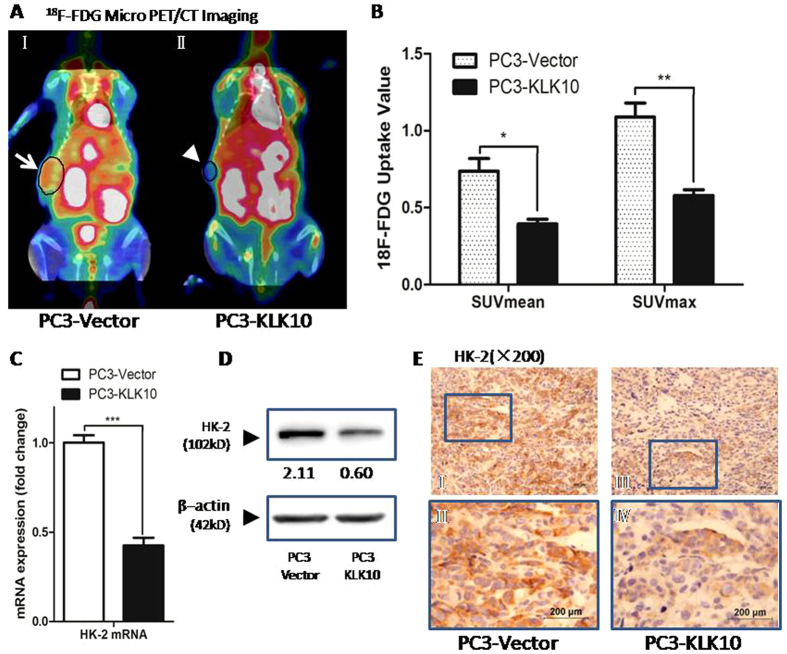
KLK10 gene inhibits the glucose metabolism of PC3 cells with the lower ^18^F-FDG uptake via the down-regulation of HK-2. (**A**: I, II) ^18^F-FDG micro PET/CT scan showed that the^18^F-FDG uptake of the transplanted tumour was lower in the PC3-KLK10 group (arrow head) than in the Vector group (arrow); in the images, hot spots of ^18^F-FDG could also be found in several organs such as the heart, liver, spleen, digestive and urinary system, but these hot spots did not disturb the imaging of the subcutaneous transplantation tumour. (**B**) Bar chart of ^18^F-FDG uptake semi-analysis showed that the uptake of ^18^F-FDG in KLK10-over-expressing PC3 transplantation tumour was low, with low SUV_mean_ and SUV_max_ (0.39 ± 0.05 vs. 0.74 ± 0.16, 0.58 ± 0.07 vs. 1.09 ± 0.18; *P* < 0.05, *P* < 0.01). (**C**) The mRNA of HK-2 was much lower in the PC3-KLK10 cell line (*P* < 0.001) than in the control group. (**D**) WB showed that the HK-2 protein was significantly down-regulated in the PC3-KLK10 cell line. (**E**: I-IV) Low expression of the HK-2 protein in the PC3-KLK10 xenograft tumour tissue measured by IHC confirmed the result of WB.

The low apoptotic index of PC3-KLK10 might not be the only reason to explain the deceleration of cell proliferation. To satisfy the demand of tumour proliferation and metastasis, cancer cells need an increase in glucose consumption because of the high rates of glycolysis, termed as ‘the Warburg effect’^19^. Enhanced glucose uptake is sufficiently prevalent such that it is taken advantage of to image cancer cells in clinical application using the glucose analogue ^18^F labelled 2-fluoro-2-deoxy-D-glucose (^18^F-FDG) by PET or PET/CT, which is available for diagnosing and staging cancer, evaluating treatment and monitoring cancer relapse for patients^20^. In our study, we utilised ^18^F-FDG micro PET/CT scan to mimic the clinical situation for monitoring the effect of KLK10 gene therapy on prostate cancer *in vivo*. As expected, the glucose metabolism of PC3-KLK10 xenograft tumour was lower than that of the PC3-Vector group. In nude mice, several organs, such as the heart, liver and spleen, could uptake high levels of ^18^F-FDG, without disturbing the imaging of the subcutaneous transplantation tumour (Fig. 5A). The uptake of ^18^F-FDG in PC3-KLK10 xenograft tumour was much lower than that in the Vector group, with lower SUV_mean_ and SUV_max_ (0.39 ± 0.05 vs. 0.74 ± 0.16 and 0.58 ± 0.07 vs. 1.09 ± 0.18, respectively; *P* < 0.05 and *P* < 0.01, respectively; Fig. 5B). As a result, the effect of proliferative inhibition of KLK10 in the PC3 cell line might be the combined result of apoptotic induction and glucose metabolism reduction.

Hexokinase-2 (HK-2), one isoform of HK, is the first enzyme of glycolysis, phosphorylating glucose into glucose-6-phosphate and preventing the molecule from leaking out of the cell^21^. HK-2 is up-regulated in cancer cells. This enzyme is an attractive target for anticancer treatment because of its important role in the glycolytic pathway and tumourigenesis^22^,^23^. In our study, the expression of HK-2 was significantly down-regulated in KLK10-expressed PC3 cells and xenograft tumour tissue (Fig. 5C–E), which was consistent with the results of ^18^F-FDG micro PET/CT scan.

### Up-regulation of KLK10 negatively regulates Bcl-2 expression and HK-2 expression

**Figure 6:**
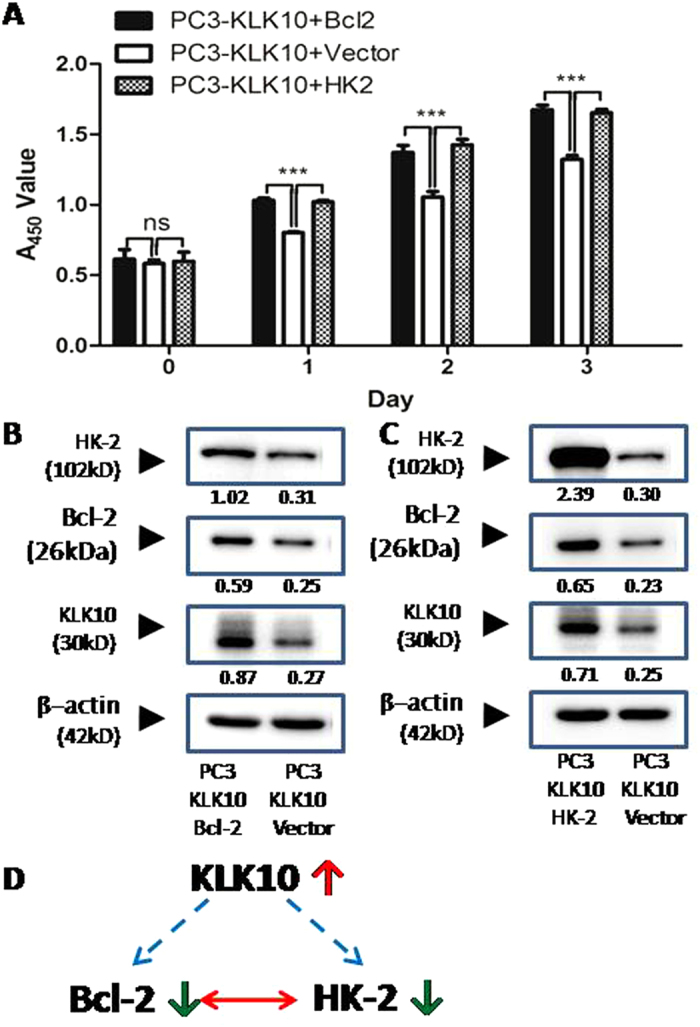
Up-regulation of KLK10 negatively regulates Bcl-2 and HK-2 expression. (**A**) CCK-8 cell proliferation assay showed that cell proliferation significantly increased in the Bcl-2- or HK-2-over-expressing PC3-KLK10 cell line compared with the PC3-KLK10 empty vector transfecting cell line (*P* < 0.001). (**B**) In the Bcl-2-over-expressing PC3-KLK10 cell line, the expression levels of both HK-2 and KLK10 increased. (**C**) In the HK-2-over-expressing PC3-KLK10 cell line, the expression levels of Bcl-2 and KLK10 also significantly increased. (**D**) Potential relationship diagram of KLK10, Bcl-2 and HK-2: the up-regulation of KLK10 might negatively regulate Bcl-2 and HK-2 expression; the expression of HK-2 and Bcl-2 might be synchronous and synergistic.

In our study, by introducing the exogenous KLK10 gene into the PC3 cell line, which demonstrated loss of KLK10 mRNA and protein expression, we observed the deceleration of PC3 cell proliferation, apoptotic induction and glucose metabolism reduction. At the molecular level, up-regulated KLK10 expression was accompanied with down-regulated expression of Bcl-2 and HK-2 mRNA and proteins. These results indicated the anticancer role of the KLK10 gene and the potential relationship among KLK10, Bcl-2 and HK-2. In the PC3-KLK10 cell line, Bcl-2 and HK-2 proteins were both down-regulated compared with those in the PC3-Vector cell line. To investigate whether the down-regulation of Bcl-2 and HK-2, which was caused by the up-regulation of KLK10, decelerates PC3 cell proliferation, we up-regulated the Bcl-2 and HK-2 genes, respectively, in the PC3-KLK10 cell line. In the Bcl-2 or HK-2 over-expressed PC3-KLK10 cell lines, cell proliferation was significantly increased compared with the PC3-KLK10 empty vector transfecting cell line (Fig. 6A). Thus, PC3 cell proliferative ability could be recovered via up-regulation of Bcl-2 or HK-2 in the exogenously KLK10 over-expressed PC3 cell line. In the Bcl-2 over-expressed PC3-KLK10 cell line, the expression levels of HK-2 and KLK10 both increased (Fig. 6B). In the HK-2 over-expressed PC3-KLK10 cell line, the expression levels of Bcl-2 and KLK10 also significantly increased (Fig. 6C).

## Discussion

KLK 10/NES1 mRNA is abundantly expressed in several organs, including the breast, ovary, prostate and testis^24^. However, the tumour-specific loss of *KLK10* expression occurs in various cancer cell lines, including those from breast and prostate^6^,^8^. The present results confirmed that KLK10 expression was down-regulated in prostate cancer tissue and cell lines, including PC3, LNCaP clone FGC and DU145. The mechanism of this tumour-specific loss characteristic was reported to be CpG island methylation, especially the exon 3 hypermethylation of the KLK10 gene^25^,^26^,^27^.

Goyal *et al.*^8^ suggested that KLK10 functions as a tumour suppressor. Zheng *et al.*^28^ found that enhancing KLK10 gene expression can decrease the proliferation of human tongue cancer cells *in vitro*. Few articles reported the role of KLK10 in prostate cancer. Our study revealed that exogenously over-expressing KLK10 can decelerate the proliferation of PC3 cells and xenograft tumour. The non-invasive assessment approach ^18^F-FLT micro PET/CT showed the lower proliferation metabolism of KLK10-over-expressing PC3 tumour tissue with the lower uptake of ^18^F-FLT. This result is consistent with the down-regulated expression of the Ki-67 protein in tumour tissue. The increased number of dead PC3-KLK10 cells, as measured by the sub-G1 population, might explain the inhibition of cell growth. TUNEL staining analysis demonstrated the occurrence of apoptosis in the PC3-KLK10 cell line. However, cleaved Caspase-3 and PARP proteins, as well as Caspase-3 enzyme activation, were undetected. This result can be attributed to the low percentage of apoptosis or to the possibility that KLK10 might induce a caspase-independent apoptosis. The exact mechanism underlying cell apoptosis should be explored in further experiments.

In the current study, the down-regulation of Bcl-2 in PC3-KLK10 cells and xenograft tumour tissue shifted our focus on the mitochondria. The mitochondria play an important role in the regulation of apoptosis, including the caspase-independent apoptosis pathway. Bcl-2 family members, including Bcl-2 and Bax, regulate the mitochondrial pathway by controlling OMM permeabilisation^29^. The Bcl-2 protein, an important regulator of programmed cell death, prevents early-stage cell apoptosis by preventing the release of cytochrome c from the mitochondria^30^,^31^,^32^. The Bax protein, an apoptotic activator that forms a heterodimer with Bcl-2, interacts with and increases the opening of the mitochondrial voltage-dependent anion channel (VDAC), which leads to membrane potential loss and cytochrome c release^30^,^31^,^32^. The Bax/Bcl-2 ratio reportedly determines whether or not a cell could undergo apoptosis^33^. In the present study, although the expression of the Bax gene did not change in PC3-KLK10 cells, the down-regulation of Bcl-2 expression and the increase in Bax/Bcl-2 protein ratio might induce the KLK10-over-expressing PC3 cells to undergo apoptosis.

However, the low apoptotic index of PC3-KLK10 might not be the only reason explaining the deceleration of cell proliferation. The most important role of the mitochondria is to generate the energy supply of most cells by utilising glucose. Sadeghi^23^ demonstrated that prostate cancer cells may metabolise glucose as an energy source to activate bioenergetic pathways; glucose and glycolysis play some indispensable roles in prostate cell growth. Thus, another potential reason for cell growth inhibition might be glucose metabolism reduction. ^18^F-FDG micro PET/CT, which noninvasively estimates the effect of KLK10 on PC3 xenograft tumour *in vivo*, revealed that the uptake of ^18^F-FDG was lower in the PC3-KLK10 xenograft tumour than in the control group. This result indicated that KLK10 reduced the glucose metabolism of PC3 cells. Hence, we focused on glucose metabolism-related enzymes. The absorbed ^18^F-FDG enters the cell via the same facilitative transporters of glucose and then undergoes phosphorylation to 2-fluoro-2-deoxy-D-glucose-6 phosphate (FDG6P) via hexokinase; FDG6P remains trapped within the cytosol, being a false substrate for all further reactions^21^,^34^,^35^. HK-2 is a hexokinase that is over-expressed in many tumours. Thus, its metabolic step can be used to monitor clinical cancers via PET or PET/CT FDG analysis^36^,^37^,^38^. In the present study, the ^18^F-FDG uptake of PC3-KLK10 tumour tissue and the expression of HK-2 synchronously decreased. This result suggested that the exogenous KLK10 gene might inhibit the glucose metabolism of PC3 via HK-2 down-regulation to inhibit PC3 cell proliferation by reducing energy supply.

Aside from being a fundamental component in glycolysis, HK-2 also participates in a survival signalling nexus by controlling cell growth and preventing mitochondrial death^39^. Previous studies suggested that around 80% of HK-2 is associated with the mitochondria^40^; HK-2 is reportedly more associated with the outer mitochondrial protein VDAC in tumour cells than in normal control cells^29^. The up-regulation and mitochondrial localisation of HK-2 may not only allow cells to use mitochondria-produced ATP for glucose phosphorylation but also exert anti-apoptotic effects by interacting with the mitochondria^41^. HK-2 maintains the integrity of the OMM and thus prevents the release of key apoptogenic molecules from the intermembrane space^36^,^41^,^42^. The dissociation of HK-2 triggers apoptosis via the subsequent release of apoptosis-inducing factor, which plays a major role in caspase-independent mitochondria-mediated cell death^41^. Silencing the HK-2 transcript could destruct the targeting tumours^37^. The underlying mechanism is the induction of significant cell death and apoptosis via the depletion of the mitochondrial membrane potential^23^. HK-2 inhibition declines the expression of Bcl-2 and promotes the apoptosis of tumour cells^43^,^44^. Thus, we hypothesised that the HK-2 and Bcl-2 genes exert a complementary effect. In the current study, the exogenous introduction of the KLK10 gene into the KLK10-deficient PC3 cell line decelerated PC3 cell proliferation, induced apoptosis and reduced glucose metabolism. At the molecular level, the up-regulation of KLK10 expression was accompanied by the down-regulation of Bcl-2 and HK-2. The up-regulation of the Bcl-2 and HK-2 genes in PC3-KLK10 cells recovered cell proliferation. In addition, HK-2 and Bcl-2 were both synergistically up-regulated in the Bcl-2- or HK-2-over-expressing PC3-KLK10 cells (Fig. 6D). The up-regulation of Bcl-2 or HK-2 increased KLK10 expression compared with the PC3-KLK10 + Vector cell line. This phenomenon might be a feedback. In other words, KLK10 expression also increased to antagonise the effect of Bcl-2 or HK-2 up-regulation in PC3-KLK10 cells. This result further supports our hypothesis that KLK10 up-regulation negatively regulates the expression of Bcl-2 and HK-2. However, this hypothesis warrants further investigation for verification.

## Conclusions

Our functional studies confirmed the important role of the KLK10 gene in the tumour suppression of the KLK10-deficient, androgen-independent prostate cancer cell line PC3. The exogenous over-expression of KLK10 in PC3 cells could decelerate tumour proliferation, which is accompanied by apoptosis induction and glucose metabolism reduction. Related proteins such as Bcl-2 and HK-2 were down-regulated and KLK10 was up-regulated in the PC3 cell line and xenograft tumour. Furthermore, the up-regulation of Bcl-2 or HK-2 in the PC3-KLK10 cell line promoted cell proliferation. In addition, HK-2 and Bcl-2 were synergistically up-regulated in the Bcl-2- or HK-2-over-expressing PC3-KLK10 cells. The up-regulation of Bcl-2 or HK-2 was accompanied by an increase in KLK10 expression, which might be a negative feedback. Basing from these results, we speculated that KLK10 up-regulation negatively regulates Bcl-2 and HK-2 expression in PC3 cells. Further experiments are needed to explore the underlying mechanism and the role of KLK10 in the KLK10-deficient, androgen-dependent prostate cancer cell line LNCaP clone FGC to understand the mechanism of KLK10 in prostate cancer and explore new potential targets for therapy.

## Methods

### Ethics statement and prostate tissue specimens

This study protocol was in accordance with the approved guidelines and was approved by the Human Ethics Committee and the Research Ethics Committee of Ruijin Hospital, Shanghai Jiaotong University School of Medicine. Samples of prostate cancer were randomly selected from prostate cancer patients, who were diagnosed and treated at the Department of Urology, Ruijin Hospital, from October 2011 to June 2014. The individuals who received hormonal deprivation therapy prior to surgery were excluded. 80 patients, including 60 prostate cancer patients, as well as 20 BPH patients as control, were selected randomly. The 60 prostate cancer patients were aged 55–85 years (median age 69 years, mean age 70 ± 7 years). And the control group, 20 BPH patients were aged 55–85 years (median age 68 years, mean age 70 ± 6 years). All the patients involved provided written informed consent.

All the prostate cancer tissue specimens were received by biopsy (40 cases) or radical prostatectomy (20 cases), and all the BPH tissues were obtained from BPH patients treated by transurethral resection of the prostate. Each prostate cancer sample and matched adjacent normal tissue were evaluated and confirmed by the Department of Pathology, Ruijin Hospital. The pathological data were obtained from a clinical database, and the sample evaluation and information recording were conducted in a double-blinded manner. Grading was performed according to the Gleason score of prostate cancer lesions, 43 cases with a high-grade Gleason score (≥7) and 17 cases with a low-grade (≤6).

### Antibodies and reagent

Rabbit anti-HK-2 polyclonal antibody, mouse anti-total and cleaved Caspase-3 antibodies was purchased from Cell Signaling Technology Company (USA). Rabbit anti-total and cleaved PARP antibody were purchased from Santa Cruz Biotechnology (USA). Rabbit anti-KLK10 monoclonal antibody; Mouse anti-Bax, mouse anti-Bcl-2 and mouse anti-Ki-67 monoclonal antibodies; anti-rabbit and anti-mouse horseradish peroxidase (HRP)-conjugated secondary antibodies were purchased from Sigma-Aldrich Company (St. Louis, MO, USA); HRP-linked β-actin was purchased from Shanghai KangChen Bio-tech Inc (China).

### Immunohistochemistry assay and analysis

To eliminate endogenous peroxidase activity, tissue sections were prepared into a 4 μm section from paraffin-embedded block and dehydrated, incubated in 3% hydrogen peroxide for 25 min, followed by using heat-mediated antigen for retrieval; 10% goat serum was introduced at room temperature for 20 min to block of nonspecific antibody sites, and primary antibody (1:100) was left in the wet box at 4 °C overnight. Then, the secondary antibody was dropped into the wet box at room temperature for 50 min of incubation; DAB staining was again visualized by hematoxylin stain and then came to normal dehydration with the cover slip sealed.

In the evaluation of results, two pathologists without knowing patients’ information were responsible for assessing the results. Regarding cell counting under microscope, three fields were randomly selected and three slides for each specimen were counted. KLK10, HK-2, Bax, and Bcl-2 positive cells were defined as in whose cytoplasma brown granules could be found; Ki-67 positive cells were defined as brown nucleus found. As for the negative control, the primary antibody was replaced with phosphate-buffered saline (PBS). The staining intensity was estimated on a four-step scale (0, 1, 2, 3). Tumors were then initially categorized according to arbitrarily predefined criteria into four groups, including completely negative, strongly positive, and two intermediate groups similarly as described previously^45^. The exact criteria for these groups were as follows: negative (no staining at all); weak (1+ staining regardless of positive cell percentages or 2+ staining of ≤30% of cells); moderate (2+ staining of >30% of cells or 3+ staining of ≤50% of cells); and strong (3+ staining of >50% of cells). The moderately positive and strongly positive were calculated into the positive group; the negative and weakly positive were calculated into the negative group.

### Cell culture

RWPE-1 (ATCC, CRL-11609, purchase from BioHermes, Wuxi, China), the non-neoplastic human prostate epithelial cell, was maintained in K-SMF (Life Technologies, USA) supplemented with 5 ng·mL^−1^ epidermal growth factor (EGF) and 50 μg·mL^−1^ bovine pituitary extract. PC3 (ATCC, CRL-1435), LNCaP clone FGC (ATCC, CRL-1740), DU145 (ATCC, HTB-81) and HEK293T (ATCC, CRL-11268) were purchased from Shanghai Institute of Cell Biology, Chinese Academy of Sciences, Shanghai, China. PC-3 and LNCaP clone FGC were cultured in RPMI 1640 medium (Bio-Whittaker Europe, Verviers, Belgium), DU145 in DMEM (Life Technologies, USA,) all supplemented with 10% fetal bovine serum (FBS), 100 units·mL^−1^ penicillin and 100 μg/mL streptomycin at 37 °C in a 5% CO_2_ atmosphere. HEK293T was cultured in DMEM with 10% FBS at 37 °C and 5% CO_2_.

### Generation of stable KLK10-expressed PC3 cell line

The KLK10 cDNA (Gene ID 5655) was amplified from a plasmid pCMV-KLK10 (reserved in our laboratory) containing the full-length KLK10 sequence, then cloned into the lentiviral vector pLVX-MCS-IRES-ZsGreens1 (purchase from Clontech) by restriction endonuclease EcoR1and BamH1 digestion and T(4) DNA ligase ligation. The primers sequences of KLK10 with EcoR1 and BamH1 were as below: KLK10_EcoR1 sense primer 5′-CCGGAATTCATGAGAGCTCCGCACCTCC-3′; KLK10_BamH1 anti-sense primer 5′-CGCGGATCCTCAGTTGGAGCGTATGACT-3′. After transformation into competent *E. coli*, the candidate clones were identified by PCR and nucleotide sequencing. The recombinant plasmid pLVX-NES1-IRES-ZsGreens1 and the two packaging plasmids (pVSVG and pCMVΔ8.91) were co-transfected into HEK293T cells by lipofectamine 2000^TM^ (Invitrogen Life Technologies, USA). Two days after transfection, strong green fluorescence was observed in HEK293T cells under fluorescent microscope. The cell supernatant, which contained recombinant lentiviral particles, was used to transduce KLK10 gene into PC3 to obtain the KLK10-overexpressing stable cell line PC3-KLK10. Fluorescent microscope was used to assess the transfection efficiency. The high-purity of target cells sorting was performed by flow cytometry, depending on the strong green fluorescence expression. Quantitative realtime PCR and western blotting was used to confirm the KLK10-overexpression mRNA and KLK10 protein. Using the same method, a high-purity blank control cell line, named as PC3-Vector, was obtained by using the pLVX blank plasmid. The cell culture of PC3-KLK10 and PC3-Vector were as same as the cell culture method of PC3.

### Transfection

After reaching 70% confluence, PC3-KLK10 cells were transfected with pCDNA-3.1-Bcl-2, pCDNA-3.1-HK-2 and pCDNA-3.1-Vector (purchased from Genscript Company, USA) respectively, using lipofectamine 2000^TM^ (Invitrogen Life Technologies, USA) according to the manufacturer’s instructions. After 4 hours’ incubation, these PC3-KLK10 cell lines were grown back into normal cell culture medium. All three cell lines were cultured as the cell culture method of PC3. Cells were harvested after 36 hours’ culture, noted as PC3-KLK10 + Bcl2, PC3-KLK10 + HK2 and PC3-KLK10 + Vector, respectively. These three cell lines’ proliferative ability was analyzed by cell proliferation assay. The Bcl-2, HK-2 and KLK10 proteins were analyzed by WB.

### RNA isolation and quantitative real-time PCR (qRT-PCR)

Total RNA was isolated using RNeasy Mini Kit (QIAGEN, Germany). After quantification, 1 μg of RNA was reverse-transcribed into first strand cDNA using AMV reverse transcriptase (TAKARA, Japan) in 20-μl reaction system. PCR (20 μl) includes 4 μl 5 × PCR buffer, 2 μl of 10 mM dNTP, 0.5 μl Ribonuclease Inhibitor, 0.1 μl of the upstream and downstream Random 9mers primers respectively, 1 μl AMV reverse transcriptase, 4 μl template cDNA, plus ddH_2_O, was complemented to 20 μl. Reaction conditions were as follows: denaturation at room temperature for 10 min and at 42 °C for 60 min.

Fluorescence quantitative real-time PCR was performed with the double-stranded DNA dye SYBR Green PCR Master Mix Reagents (Applied Biosystems, Foster City, CA, USA) using the ABI PRISM® 7300 Real-Time PCR system (Applied Biosystems, Foster City, CA, USA) according to the manufacturer’s instructions. A 10-μL PCR mixture was prepared containing3 μL of cDNA, 5 μL SYBR Green PCR Master Mix, 0.2 μL of each primer and 1.6 DEPC H_2_O to complement to 10 μL. Reaction conditions were as follows: The temperature cycles to optimal reaction was at 50 °C for 2 min and at 95 °C for 10 min to activate the PCR; followed by 40 cycles of 95 °C for 30 s, 60 °C for 60 s, and 72 °C for 35 s. The plate reading was taken after extension at 72 °C and a melting curve was obtained by performing a thermal cycle of 95 °C for 30 s, 60 °C for 30 s, and 72 °C for 45 s. The reactions were carried out in a 96-well optical reaction plates or MicroAmp reaction tubes (Applied Biosystems, Foster City, CA, USA). All data was analyzed by ABI Prism 7300 SDS Software (Applied Biosystems, Foster City, CA, USA) to obtain the Ct (threshold cycle) value. All samples were tested with the reference gene β-actin for data normalization to correct for variations in RNA quality and quantity. The specific oligonucleotide primers used were shown in Table 2. All experiments were performed in triplicate.

### Western blotting

Cells were harvested, washed with ice-cold PBS, and lysed with lysis buffer (62.5 mM Tris-HCl, pH 6.8, 100 mM DTT, 2% SDS, 10% glycerol). Protein extracts were equally (10 μg) loaded on 12% SDS-polyacrylamide gel (PAGE), electrophoresed, and transferred to an Immobilon–polyvinylidene fluoride (PVDF) membrane (Schleicher & Schuell, Dassel, Germany). After blocking with 5% nonfat milk in PBS, the membranes were incubated with the following primary antibodies respectively at 4 °C overnight: KLK10, HK-2, Bax, Bcl-2, total and cleaved PARP, total and cleaved Caspase-3 (1:1000), HRP-linked β-actin (1:5000); and followed by the corresponding horseradish peroxidase (HRP)-conjugated secondary antibodies (1:2000). Beta-actin was used as loading control. All experiments were repeated three times with similar results. Bands were detected by chemiluminescence using a Pierce ECL Western Blotting Substrate (Thermo Scientific Pierce, USA) and recorded by an Image Quant LAS-4000 Luminescent Image Analyzer (GE Healthcare, Piscataway, NJ, USA). Quantification of western blotting bands was performed using ImageJ2 × (2.1.4.7) software and normalized to normalizator signal (β-actin).

### Cell proliferation assay

Cells were seeded (5000 cells/well) into 96-well flat plates and incubated for 3 consecutive days at 37 °C in 5% CO_2_ atmosphere. Cellular proliferation was assessed at the moment of cell attachment, about 7 hours after cells were plated, then at 24 h, 48 h, and 72 h, using a cell counting kit (CCK-8)(Dojindo, Kumamoto, Japan), according to manufacturer’s instructions. After co-cultured for 4 h, the absorbance was detected at a wavelength of 450 nm and adjusted at a wavelength of 690 nm. Each experiment was conducted in triplicate and repeated three times.

### Cell cycle assay

Cell cycle distributions were analyzed utilizing propidium iodide (PI; Sigma-Aldrich Company, St. Louis, MO, USA) staining with flow cytometry. Cells were seeded at a density of 2.5 × 10^5^ cells/well in 6-well flat plate and incubated overnight. Then cells were harvested and fixed with 70% cold ethanol at −20 °C overnight, washed with PBS, treated with RNase A (Sigma-Aldrich Company, St. Louis, MO, USA) and PI in the dark for 30 min at room temperature. Samples were analyzed using flow cytometry (BD FACS Calibur; BD Biosciences, USA). The percentage of cells in the sub-G1, G0/G1, S, and G2/M phases of the cell cycle were analyzed with BD CellQuest Pro Software (BD Biosciences, USA). Experiments were repeated three times, in triplicate for all cell lines.

### TUNEL

PC3-KLK10 and PC3-Vector cells were seeded (2 × 10^5^ cells/well) onto clean cover class in 6-well flat plate and incubated for 1 night at 37 °C in 5% CO_2_ atmosphere. After cell climbing piece, cells were fixed in 4% paraformaldehyde for 1 hour at room temperature, then were washed with cold 1 × PBS softly for three times. The apoptosis was detected *in situ* by a commercial TUNEL assay kit (Roche, Shanghai, China). The TUNEL assay was performed following the manufacturer’s protocol. The apoptosis was analyzed by counting the positive cells (brown-stained), and total number of cells in five randomly selected fields at ×400 magnification. The percentage of positive cells, namely apoptotic index, was calculated as: the number of positive cells/total number of nucleated cells ×100%. Experiments were repeated three times, in triplicate for all cell lines.

### Caspase-3 enzyme activity assay

Cells (2 × 10^6^ cells) were harvested and treated with 100 μl lysis lotion for 15 min on ice. After centrifugation, the supernatant of samples were collected in cold centrifuge tube, treated with lotions in the Caspase-3 activity assay kit (Beyotime, China), and incubated at 37 °C overnight. Then the intensity of the color was measured at 450 nm using a spectrophotometer. Calibration standard was assayed at the same time and allowed to produce a standard curve of Optical Density (OD) versus caspase-3 concentration. The concentration of caspase-3 in the samples was then determined by comparing the OD of the samples to the standard curve. All were processed strictly according to the manufacturer’s instruction. Experiments were repeated three times, in triplicate for all cell lines.

### Animal experiments

Male nude mice (4 weeks old) were purchased from the Experimental Animal Center, Shanghai Jiaotong University School of Medicine. All animal experiments protocols were performed in accordance with relevant guidelines and were approved by the Animal Care and Use Committee of Shanghai Jiaotong University School of Medicine (Shanghai, China). The mice were randomly divided into 2 groups of 5 mice each, named as PC3-KLK10 and PC3-Vector groups. Target cells were suspended in sterile PBS, and 5 × 10^6^ cells (0.1 ml) were injected into the subcutaneous of right flank of the nude mice. Every 4 days the vital signs were examined, as well as the mice weight and the tumor size were recorded, including the long (a) and short (b) diameter of tumor. The volume formula: volume [mm^3^] = ab^2^/2. It lasted for 16 days until difference of the tumor size for each group was apparent. After the ^18^F-FDG micro PET/CT imaging of PC3-KLK10 and PC3-Vector groups, the mice were sacrificed by cervical dislocation, and the tumors were harvested. Then, the tumor tissue was bar cutting into almost 1 mm^3^, immobilized in 100% paraformaldehyde, embedded and chipped, tested the expression of KLK10, Ki-67, HK-2, Bax and Bcl-2 protein by immunohistochemisty assay.

### ^8^F-FLT and ^18^F-FDG micro PET/CT imaging

All mice were undergone the ^18^F-FLT micro PET/CT imaging on the Siemens Inveon Micro PET/CT (Inveon MM Platform, Siemens Preclinical Solutions, Knoxville, Tennessee, USA) with a computer-controlled bed and 8.5 cm transaxial and 5.7 cm axial fields of view. Mice were anesthetized with 3% Isoflurane in O_2_ gas for ^18^F-FLT tail intravenous injection (a single injection of 0.2 ml with an activity of 5.3–6.4 MBq). Waiting for 30 min post injection, mice were first CT scanned for 20 min and then continuously PET scanned for 60 minutes. Mice were anesthetized with 1.5% Isoflurane during scanning. Inveon Acquisition Workplace (IAW) was used for scanning process. The CT data was used for both scatter and attenuation correction. Images were reconstructed by an OSEM3D (Three-Dimensional Ordered Subsets Expectation Maximum) algorithm followed by MAP (Maximization/Maximum a Posteriori) or Fast MAP provided by IAW. The 3D regions of interest were drawn over the tumor tissue guided by CT images and tracer uptake was measured using the software of Inveon Research Workplace 3.0. Individual quantification of the ^18^F-FLT uptake in each of them was calculated to get the SUV_mean_ and SUV_max_.

The third day, all mice were undergone the ^18^F-FDG micro PET/CT imaging on the same machine with the same imaging procedure, the same manipulation parameters, as well as the same reconstructed and analysis methods, except for the mice preparation that all mice were starved for at least 8 hours, as well as the ^18^F-FDG tail intravenous injection 30 min before imaging (a single injection of 0.2 ml with an activity of 5.3–6.4 MBq).

### Statistical analysis

Statistical analysis was performed using GraphPad Prism 5.0 software. Pearson chi-square (χ^2^) tests were used for human tissue KLK10 expression comparisons. Statistical comparisons between two groups were performed using Student’s *t* tests. A value of p < 0.05(*) was considered statistically significant, p < 0.01(**) and p < 0.001(***) considered remarkably significant, p > 0.05(ns) considered no difference. Data were presented as the mean with standard deviation (SD) in the line or bar graphs.

## Additional Information

**How to cite this article**: Hu, J. *et al.* NES1/KLK10 gene represses proliferation, enhances apoptosis and down-regulates glucose metabolism of PC3 prostate cancer cells. *Sci. Rep.* **5**, 17426; doi: 10.1038/srep17426 (2015).

## Supplementary Material

Supplementary Information

## Figures and Tables

**Table 1:** Expression of KLK10 protein in BPH tissue, normal tissue adjacent to the prostate cancer and tissue of prostate cancer.

Groups	Case	KLK10 expression				Positive Rate (%)	χ2 Value	P Value
		−	+	+ +	+++			
BPH tissue	20	0	0	2	18	100	60.66	<0.0001^a^
NTAPC	60	0	4	15	41	93.3	64.28	<0.0001^b^
CTPC	60	19	18	21	2	38.3		

Abbreviation: BPH: benign prostate hyperblastosis; NTAPC: normal tissue adjacent to the prostate cancer; CTPC: cancer tissue of prostate cancer.

^a^comparison between BPH and prostate cancer tissue.

^b^comparison between the normal tissue adjacent to the prostate cancer and the cancer tissue.

**Table 2:** Gene-specific Primers for realtime-PCR.

Name	Orientation	Sequence of Primer
KLK-10	Sense	ACTGCGGAAACAAGCCACT
	Anti-sense	GGTACTTGGGATGGACAACAGA
Bcl-2	Sense	ATGACTGAGTACCTGAACCGGC
	Anti-sense	GAGACAGCCAGGAGAAATCAAAC
Bax	Sense	CCTTTTGCTTCAGGGTTTCAT
	Anti-sense	CATCCTCTGCAGCTCCATGTTA
HK-2	Sense	AAGGCTTCAAGGCATCTG
	Anti-sense	CCACAGGTCATCATAGTTCC
β-actin	Sense	AGGCACCAGGGCGTGAT
	Anti-sense	GCCCACATAGGAATCCTTCTGAC
