# Supplementary material for: NES1/KLK10 gene represses proliferation, enhances apoptosis and down-regulates glucose metabolism of PC3 prostate cancer cells
Source: Sci Rep. 2015 Nov 30;5:17426. doi: 10.1038/srep17426 (PMC4663538; doi:10.1038/srep17426)

NES1/KLK10 gene represses proliferation, enhances apoptosis and down-regulates glucose metabolism of PC3 prostate cancer cells

Running Title: NES1/KLK10 suppress PC3

Jiajia Hu[1], Hu Lei[2], Xiaochun Fei[3], Sheng Liang[4], Hanzhang Xu[2], Dongjun Qin[2], Yue Wang[1], Yingli Wu[2*], and Biao Li[1*]

Supplemental figure legends:

Supplemental figure 1

(A: I-IV) Bax protein expression in PC3-KLK10 and PC3-Vector xenograft tissues measured by immunohistochemistry was similar. (B and C) WB showed that the cleaved Caspase-3 and PARP proteins in PC3-KLK10 cell line were not detected. (D: I-II) Plots and data of flow cytometry in PC3-KLK10 and PC3-Vector cell lines for Figure 4-A.

Supplemental figure 2

(A) Marker of western blotting. (B) Full-length blots of KLK10 protein in RWPE-1, PC3, DU145, LNCaP clone FGC for Figure 1-C; full-length blots of KLK10 protein in PC3, PC3-Vector and PC3-KLK10 for Figure 2-B. (C) Full-length blots of Bcl-2 protein in PC3, PC3-Vector and PC3-KLK10 for Figure 4-D. (D) Full-length blots of Bax protein in PC3-Vector and PC3-KLK10 for Figure 4-D. (E) Full-length blots of HK-2 protein in PC3-Vector and PC3-KLK10 for Figure 5-D. The β-actin protein is as same as one in (D).

Supplemental figure 3

(A) Full-length blots of total and cleaved Caspase-3 protein in PC3-Vector and

PC3-KLK10. The cleaved Caspase-3 protein in PC3-KLK10 was not detected. (B) Full-length blots of total and cleaved PARP protein in PC3-Vector and PC3-KLK10. The cleaved PARP protein in PC3-KLK10 was not detected. (C) Full-length blots of HK-2 protein in PC3-KLK10+HK-2 and PC3-KLK10+ Vector for Figure 6-C. (D) Full-length blots of KLK10 protein in PC3-KLK10+HK-2 and PC3-KLK10+Vector for Figure 6-C. (E) Full-length blots of Bcl-2 protein in PC3-KLK10+HK-2 and PC3-KLK10+Vector for Figure 6-C. (F) Full-length blots of Bcl-2 protein in PC3-KLK10+Vector and PC3-KLK10+Bcl-2 for Figure 6-B. (F) Full-length blots of HK-2 protein in PC3-KLK10+Vector and PC3-KLK10+Bcl-2 for Figure 6-B. (G) Full-length blots of KLK10 protein in PC3-KLK10+Vector and PC3-KLK10+Bcl-2 for Figure 6-B.

Supplemental figure 1:

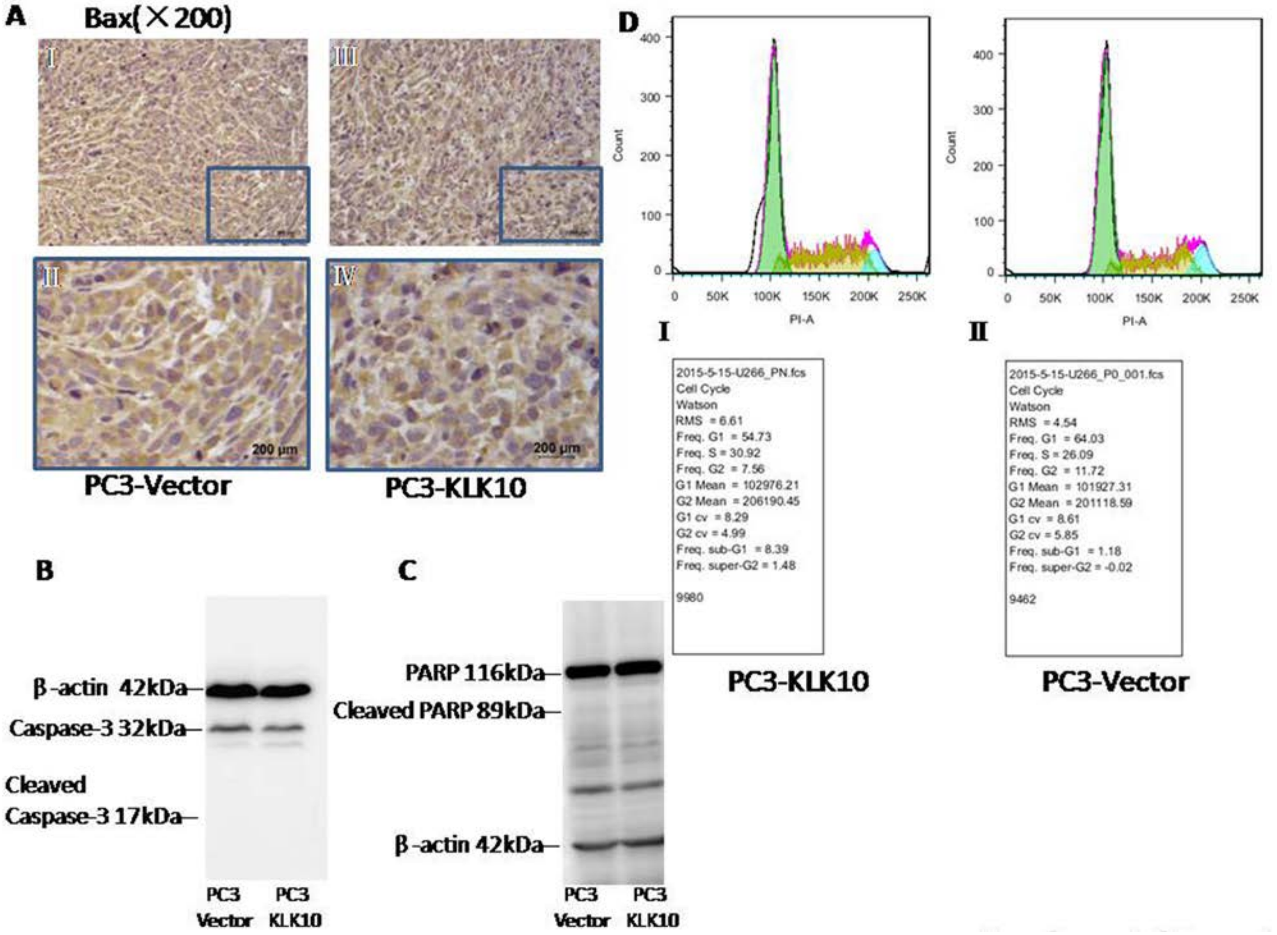

Supplemental figure 2

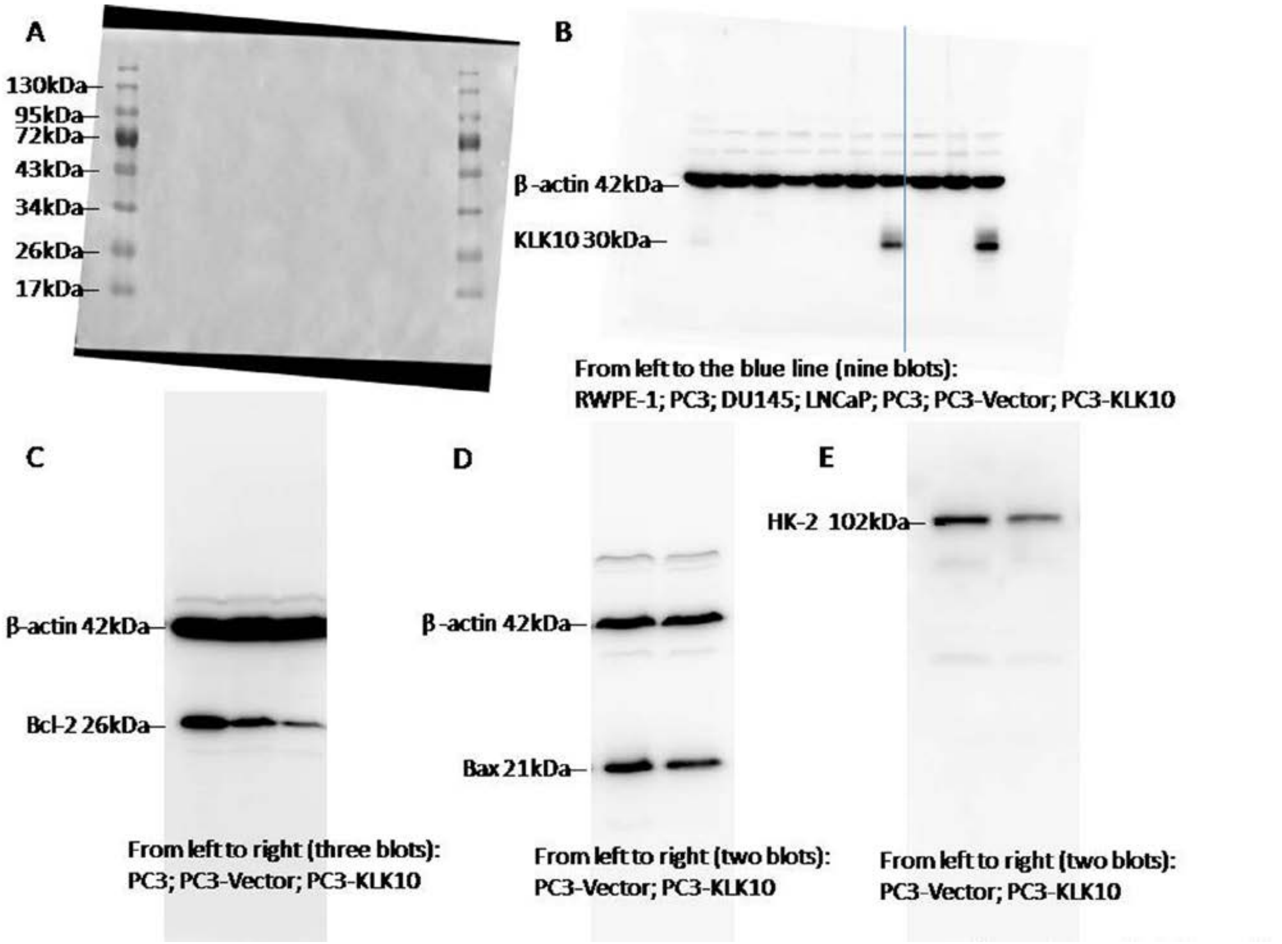

Supplemental figure 3

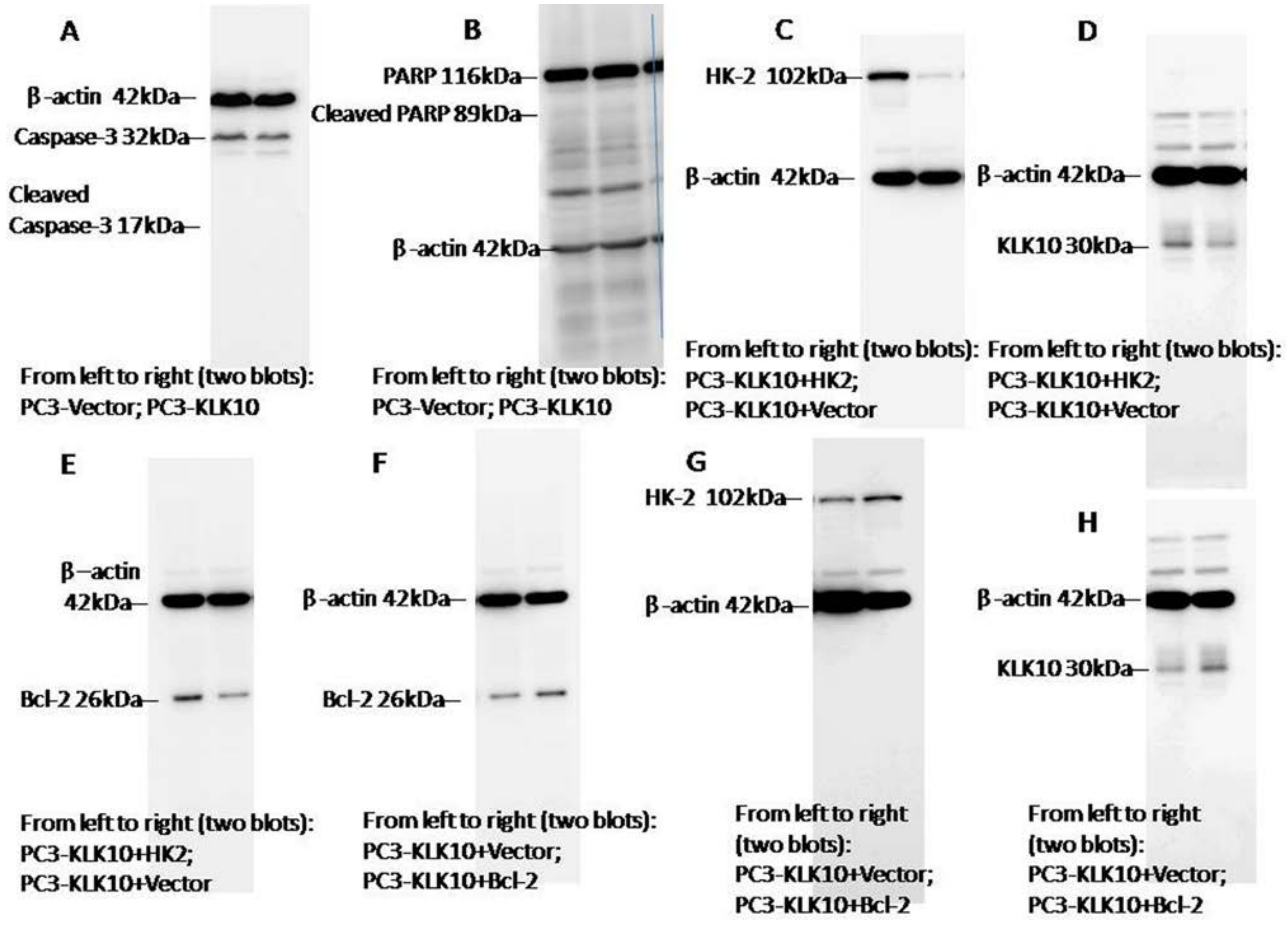

Supplement: Supplementary Information [file srep17426-s1.pdf]
